# Chemoresistant colorectal cancer cells and cancer stem cells mediate growth and survival of bystander cells

**DOI:** 10.1038/bjc.2011.449

**Published:** 2011-11-01

**Authors:** D Bose, L J Zimmerman, M Pierobon, E Petricoin, F Tozzi, A Parikh, F Fan, N Dallas, L Xia, P Gaur, S Samuel, D C Liebler, L M Ellis

**Affiliations:** 1 Department of Surgical Oncology, The University of Texas MD Anderson Cancer Center, Houston, TX 77230, USA; 2 Department of Cancer Biology, The University of Texas MD Anderson Cancer Center, Houston, TX 77230, USA; 3 Jim Ayers Institute for Precancer Detection and Diagnosis, Vanderbilt-Ingram Cancer Center, Vanderbilt University, Nashville, TN 37232, USA; 4 Center for Applied Proteomics and Molecular Medicine, George Mason University, Fairfax, VA 22030, USA

**Keywords:** chemoresistance, colorectal cancer, chemotherapy, conditioned medium, cancer stem cells

## Abstract

**Background:**

Recent studies suggest that cancer stem cells (CSCs) mediate chemoresistance, but interestingly, only a small percentage of cells in a resistant tumour are CSCs; this suggests that non-CSCs survive by other means. We hypothesised that chemoresistant colorectal cancer (CRC) cells generate soluble factors that enhance survival of chemonaive tumour cells.

**Methods:**

Chemoresistant CRC cells were generated by serial passage in oxaliplatin (Ox cells). Conditioned media (CM) was collected from parental and oxaliplatin-resistant (OxR) cells. CRC cells were treated with CM and growth and survival were assessed. Tumour growth rates were determined in nude mice after cells were treated with CM. Mass spectrometry (MS) identified proteins in CM. Reverse phase protein microarray assays determined signalling effects of CM in parental cells.

**Results:**

Oxaliplatin-resistant CM increased survival of chemo-naive cells. CSC CM also increased growth of parental cells. Parental and OxR mixed tumours grew larger than tumours composed of parental or OxR cells alone. Mass spectrometry detected unique survival-promoting factors in OxR CM compared with parental CM. Cells treated with OxR CM demonstrated early phosphorylation of EGFR and MEK1, with later upregulation of total Akt .We identified progranulin as a potential mediator of chemoresistance.

**Conclusion:**

Chemoresistant tumour cells and CSCs may promote resistance through soluble factors that mediate survival in otherwise chemosensitive tumour cells.

Resistance to chemotherapy represents the major obstacle to survival in patients with metastatic chemoresistant colorectal cancer (mCRC; Dy *et al*, 2009). Despite the addition of targeted therapies to chemotherapy, the outcomes for patients with mCRC have only modestly improved (Hurwitz *et al*, 2004; Beatty and Giantonio, 2008; Saltz *et al*, 2008; Goldberg *et al*, 2009; Punt and Tol, 2009). Cancer stem cells (CSCs) have been hypothesised to mediate chemoresistance within the tumour mass (Cho and Clarke, 2008; Pohl *et al*, 2008; Visvader and Lindeman, 2008; Ricci-Vitiani *et al*, 2009). Colorectal CSCs comprise a small sub-population of tumour cells and have been also been referred to as tumour-initiating cells (O’Brien *et al*, 2007; Ricci-Vitiani *et al*, 2007; Barker *et al*, 2009; Zhu *et al*, 2009). Chemoresistant colorectal cancer stem cells from tumour samples exhibit resistance to treatment with chemotherapeutic agents such as oxaliplatin and 5-fluorouracil (5-FU), possibly due to drug efflux, autocrine survival signalling, and alterations in DNA damage repair mechanisms (Sussman *et al*, 2007; Todaro *et al*, 2007). In cell culture systems, chronic exposure to chemotherapy selects for a chemoresistant phenotype that demonstrates properties consistent with the CSC phenotype (Yang *et al*, 2006; Dallas *et al*, 2009). Although cells that are intrinsically resistant to chemotherapy and CSCs comprise small minorities of cells within a tumour, a relatively larger percentage of viable tumour cells remain after chemotherapy (Blazer *et al*, 2008). As an explanation for how a small fraction of cells may influence tumour response to treatment, we hypothesised that resistant CRC cells and CRC stem cells are able to mediate growth and survival of nearby cells by means of soluble factors that are released into the tumour microenvironment.

A pharmacological perspective on mechanisms of resistance to chemotherapy in CRC focuses on mechanisms intrinsic to cancer cells, specifically on biochemical mechanisms by which the effects of such drugs as 5-FU and oxaliplatin are overcome (Viguier *et al*, 2005; Bhushan *et al*, 2009). However, few models of resistance to oxaliplatin exist to provide a clear mechanistic understanding of how resistance is created in tumours, which initially respond to oxaliplatin (acquired resistance), an event that may be distinct from resistance based on a germline variation or a mutation that has already occurred in tumour cells (primary resistance). There are data to suggest mechanisms for ERCC1 and XRCC1 dysregulation leading to resistance to chemotherapy. (Prewett *et al*, 2007), for example, found that cetuximab (EGFR antibody) overcomes oxaliplatin resistance in mice, and that cetuximab reduces expression of ERCC1, XPF, and XRCC1 in treated cells. However, EGFR-targeted monoclonal antibodies does not significantly improve the efficacy of oxaliplatin-based regimens, suggesting that other, yet unidentified, factors have a role in chemoresistance in patients with mCRC.

Progranulin is a secreted glycoprotein that is thought to be involved in the pathogenesis of frontotemporal lobar dementia, and therefore progranulin has been most intensively investigated in its role in neurobiology. These investigations have led to the very recent discovery that progranulin associates with cell surface sortilin, a single transmembrane domain receptor that is also a co-receptor for neurotensin and is a member of a family of vacuolar sorting proteins (Vps10). Hu *et al* (2010) have recently shown that association of progranulin with sortilin results in endocytosis and lysosomal degradation of progranulin. Sortilin appears to participate in signal transduction pathways that promote apoptosis of neurons, whereas progranulin is generally associated with neuron growth and survival, suggesting that interaction with progranulin may function to inhibit sortilin-mediated cell death. Progranulin has been implicated in the pathogenesis of ovarian cancer, and it may be a marker of poor prognosis (Han *et al*, 2010). No role for progranulin has been identified in colorectal cancer, but sortilin has been demonstrated to be expressed by HT29 human colorectal cancer cells and may modulate response to nerve growth factor in these cells (Martin *et al*, 2002; Navarro *et al*, 2002; Morinville *et al*, 2004). The results presented here suggest that progranulin may have a role in colorectal cancer as a soluble factor that mediates growth and chemoresistance.

The role of soluble factors in the development of resistance remains unclear. Our previous work on cell lines resistant to oxaliplatin and 5-FU suggests that multiple signalling pathways may be involved in the development of acquired resistance, including those involving insulin-like growth factor receptor and c-Src (Yang *et al*, 2006; Dallas *et al*, 2009; Kopetz *et al*, 2010). In this study, we asked whether sub-populations of tumour cells (chemoresistant CRC cells and CRC stem cells) within a tumour mass secrete soluble factors that engage intracellular signalling pathways and mediate chemoresistance of neighbouring tumour cells. Our study has two clinically relevant implications related to the nature of acquired resistance. The first is that resistance to chemotherapy may be only partially overcome by targeting survival pathways in resistant cells themselves when multiple mechanisms may be at work in both resistant and bystander tumour cells. The second is that chemoresistant cells are not all CSCs, suggesting that CSC-directed therapies might also be inadequate to achieve durable complete responses.

## Materials and methods

### Cell culture reagents

Cell lines (American Type Culture Collection, Manassas, VA, USA) were maintained in culture using standard protocols in MEM supplemented with 10% fetal bovine serum, vitamins, nonessential amino acids, penicillin-streptomycin, sodium pyruvate, and L-glutamine (Life Technologies, Grand Island, NY, USA). Unless otherwise specified, chemotherapeutic agents were used as follows: oxaliplatin at 2 *μ*M, 5-FU at 2 *μ*g ml^−1^.

### Development of chemoresistant cell lines

Human CRC cell lines HT29, HCT116, RKO, and KM12L4 were used to generate chemoresistant lines by chronic exposure to increasing concentrations of oxaliplatin or 5-FU as previously described (Yang *et al*, 2006; Dallas *et al*, 2009). HT29, HCT116, and RKO cells were from the American Type Culture Collection. KM12L4 were provided by the courtesy of IJ Fidler, MD Anderson Cancer Center. Cells were exposed to increasing levels of chemotherapeutic agents oxaliplatin and 5-FU, until cells were growing at clinically relevant levels of drug, that is, 2 *μ*M for oxaliplatin and 2 *μ*g ml^−1^ for 5-FU. Resistant cell lines were maintained under constant treatment with drug, except during trypsinisation and passaging of cells at which point drug was added the day after replating.

### Enrichment for CSCs

Cultures enriched for CSCs were achieved by two different methods. To enrich for CSCs and sort cells based on the side-population assay, cells that exhibited the ability to exclude Hoechst 33342 dye were sorted and cultured as previously described (Goodell *et al*, 1996; Ho *et al*, 2007). The Aldefluor assay was used to sort cells based on the ALDH1A1 activity according to the manufacturer's instructions (Stemcell Technologies, Vancouver, British Columbia, Canada). To generate conditioned media (CM), cells were then cultured to ∼40–50% confluence, at which point standard culture media were removed and serum-free media were added to generate CM as described below.

### Generation of CM

Parental, chemoresistant, and CSC-enriched cell cultures were seeded such that 48 h would pass between trypsinisation and the initiation of CM production. In the case of chemoresistant cell lines, drug treatment was initiated 24 h after splitting cells. After another 24 h, media were removed and cells were washed three times with PBS. After washes, cells were incubated with DMEM supplemented in standard manner with essential amino acids, antibiotics, and vitamins but without FCS. Cells were then incubated for 48 h – the resulting media were drawn off and used as CM after centrifugation at 1500 **g** for 10 min to remove debris.

### Conditioned media experiments

*In vitro* CM experiments were conducted by culture of parental cells in 96-well plates such that optical density (OD) by MTT assay (described below) would read approximately 0.1 after 48 h. At this point, media were removed from the wells and replaced with CM generated as described above. Six wells were used for each experimental condition, and multiple plates were treated for assay at several time points as indicated. One plate in each experimental run was immediately assayed in order to establish the baseline OD and ensure uniformity of plating across the 96-well plate. *In vitro* proliferation was assessed with tetrazolium salt MTT as described earlier (Grey *et al*, 2005). Assays were carried out by incubating each plate with 20 *μ*l of MTT substrate for 1 h followed by removal of medium and addition of 200 *μ*l of DMSO. Plates were read at a wavelength of 570 nm, with OD reported relative to blank wells containing only DMSO.

### Progranulin and chemoresistance experiments

*In vitro* growth and chemoresistance experiments were conducted by culture of parental cells in 96-well plates such that OD by MTT assay (described above) would read approximately 0.1 after 48 h. At this point media were removed from the wells and replaced with media containing 1 *μ*g ml^−1^ recombinant human progranulin (R&D Systems, Minneapolis, MN, USA) and/or oxaliplatin at concentrations indicated on the *x*-axes or in figure legend (Figure 5). *In vitro* proliferation was assessed by MTT assay as described above (Grey *et al*, 2005).

### 5-FU-resistant (5-FU-R) cells

HT29 and HCT116 cells were made resistant to 5-FU as described above. Conditioned media from 5-FU-R cells were used in *in vitro* growth and chemoresistance as described above. On the basis of negative results (data not shown), 5-FU-R HT29 cells were used as an additional control for our mass spectrometric (MS) characterisation of the CM. 5-FU-R cells were not used in *in vivo* experiments.

### Generation of luciferase-labelled cells and cell-mixing experiments

HT29 cells were stably transfected with the luciferase gene by lentiviral infection as described previously (Arumugam *et al*, 2006). In addition, these cells were stably co-transfected with the *DsRed* gene by lentiviral infection (pLVX-DsRed, Clontech, Mountain View, CA, USA). Luciferase activity in whole-cell lysates was assessed using a standard luminometer. Cells subsequently were sorted for high expression of DsRed by fluorescence-activated cell sorting. Sorted cells were then passaged for use in cell-mixing experiments by plating in designated proportions with oxaliplatin-resistant (OxR) HT29 cells in a 96-well plate for luciferase activity measurement.

### *In vivo* studies

The *in vivo* cell mixing experiment was performed in nude mice, with tumours generated by subcutaneous injection of designated proportions of OxR and parental HT29 cells (100% OxR cells; 10% OxR cells, 90% parental HT29 cells; 50% OxR cells, 50% parental cells; or 100% parental cells) for a total of 10^6^ cells per injection. Male athymic nude mice, 6–8 weeks old, were obtained from the National Cancer Institute-Frederick Cancer Research Facility (Frederick, MD, USA) and acclimated for 2 weeks. All animal studies were conducted under the approved guidelines of the Animal Care and Use Committee of MD Anderson Cancer Center. Tumour growth was monitored by caliper measurement by a single investigator. Tumour volume was calculated as ((length/2) × (width^2^))±s.e.m. At the time of killing by CO_2_ asphyxiation, tumours were excised, weighed, and photographed.

For the dual-flank study, either parental or OxR cells were injected into the left flank of 10 nude mice in each arm, and these were designated as the ‘feeder’ tumours. When average ‘feeder’ tumour sizes had reached 0.1 cm^3^ for HT29 ‘feeder’ tumours, 10^6^ parental HT29 cells were injected into the right flank of each mouse, and these were designated as the ‘secondary’ tumours. Owing to slow growth, OxR ‘feeder’ tumours were given an additional week to grow before injection of 10^6^ HT29 cells in the right flank of each mouse to generate ‘secondary’ tumours. Both feeder and secondary tumours were monitored for growth.

### Mass spectrometric analysis of proteins in CM

Three 400 *μ*l aliquots of the each of the CM from the HT29, OxR, and 5-FU-R cells were evaporated *in vacuo*, resuspended in LDS–PAGE sample loading buffer and reduced with dithiothreitol (DTT) just before heating in a 70 °C water bath for 10 min. After cooling, each sample was loaded in three portions into separate lanes on an SDS–PAGE gel and the proteins were electrophoresed using a 4–12% Bis-Tris gel with MES as the running buffer. A total of three gels were processed, each have triplicate lanes of each type of CM. Each lane was cut into 17 gel pieces, which then were subjected to in-gel digestion. Briefly, the gel regions were excised and washed with 100 mM ammonium bicarbonate for 15 min. The liquid was discarded and replaced with fresh 100 mM ammonium bicarbonate and the proteins reduced with 5 mM DTT for 20 min at 55 °C. After cooling to room temperature, iodoacetamide was added to 10 mM final concentration and samples were placed in the dark for 20 min at room temperature. The solution was discarded and the gel pieces were washed with 50% acetonitrile/50 mM ammonium bicarbonate for 20 min, followed by dehydration with 100% acetonitrile. The liquid was removed and the gel pieces were completely dried, re-swelled with 0.15 *μ*g of modified trypsin (Promega, Madison, WI, USA) in 100 mM NH_4_HCO_3,_ and digested overnight at 37 °C. Peptides were extracted by three changes of 60% acetonitrile/0.1% TFA, and all extracts were combined and dried *in vacuo*. Samples were reconstituted in 25 *μ*l 0.1% formic acid before LC-MS/MS analysis on a Thermo LTQ XL ion trap mass spectrometer (Thermo Scientific, West Palm Beach, FL, USA) (Slebos *et al*, 2008). Tandem MS were acquired using a data dependent scanning mode, in which one full MS scan (m/z 400–2000) was followed by five MS/MS scans. Tandem MS were searched against the IPI human database version 3.37 using the MyriMatch algorithm version 1.12 (Tabb Lab, Vanderbilt University, Nashville, TN, USA); http://fenchurch.mc.vanderbilt.edu/software.php. The database contained both the forward as well as reversed sequences to allow for estimation of false discovery rates. Identifications were then filtered using the IDPicker algorithm using a false-positive ID threshold (default is 0.05 or 5% false positives) based on reverse sequence hits in the database, and proteins were also required to have at least two distinct peptide sequences (Zhang *et al*, 2007; Ma *et al*, 2009). Spectral count data sets were compared with the QuasiTel programme (Liebler Lab, Vanderbilt University, Nashville, TN, USA), which estimates significant differences in peptide and protein expression with a model based on a modified Poisson distribution.

### Protein pathway activation mapping analysis

Pathway activation mapping was performed using reverse phase protein microarrays (RPMA) (Paweletz *et al*, 2001) and recently used to map signalling events in CRC (Gulmann *et al*, 2009; Pierobon *et al*, 2009). Cell lysates were prepared after cells were treated with CM for indicated times and subsequently analysed by RPMA as described (Pierobon *et al*, 2009). Briefly, cellular lysates were printed on nitrocellulose-coated slides (Whatman, Inc., Sanfort, ME, USA) using a 2470 AushonArrayer (Aushon BioSystems Inc., Billerica, MA, USA) outfitted with 350 *μ*m pins. Each sample was printed in triplicate and in two-point dilution curves. Protein normalisation was performed as described previously (Pierobon *et al*, 2009) using Sypro Ruby Protein Blot Stain (Molecular Probes, Eugene, OR, USA) and visualised with NovaRay Image Acquisition Software (Alpha Innotech, San Leandro, CA, USA) to determine the total protein concentration. Before antibody staining, the slides were treated with Reblot antibody stripping solution (Chemicon, Temecula, CA, USA), and incubated for 5 h at room temperature in blocking solution with constant shaking. Blocked slides were stained with antibodies targeting total and phosphorylated proteins using an automated stainer (DakoCytomation, Carpinteria, CA, USA). Each antibody was subjected to rigorous validation for single-band specificity at the correct molecular weight by Western blotting along with the use of appropriate ligand-induction controls for phospho-specific antibodies. Catalysed Signal Amplification System kit (DakoCytomation) and fluorescent IRDye 680 Streptavidin (LI-COR Inc., Lincoln, NE, USA), were used as detection system. Stained slides were scanned with NovaRay Image Acquisition Software (Alpha Innotech). Acquired images of each slide were analysed using MicroVigene software (Vigenetech, Carlisle, MA, USA) that finds spots, performs local background subtraction, averages replicates and normalises each sample for the total protein value, and produces a final numeric relative intensity value.

## Results

### Effect of CM on cell growth and survival

**Figure 1:**
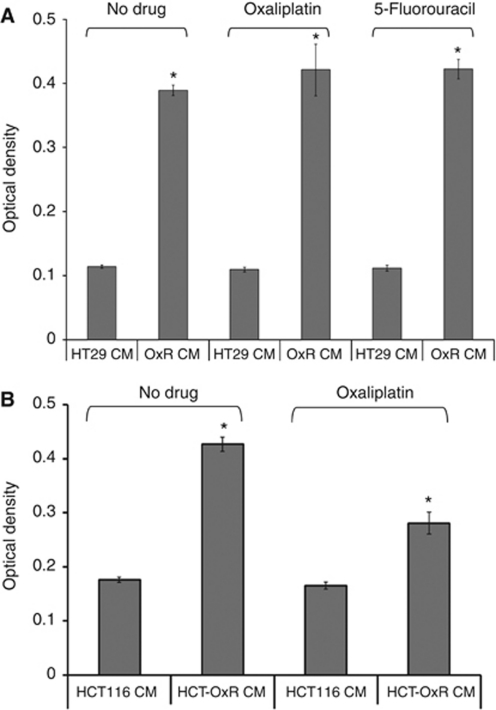
Effect of CM from OxR cells on CRC cell proliferation and survival. (**A**) Proliferative effect of CM from OxR HT29 cells. MTT assay on HT29 cells treated with CM from parental HT29 cells (HT29 CM) or from OxR HT29 cells (OxR CM). Cells were either untreated or treated with oxaliplatin as described in the Materials and Methods section. The *y*-axis displays OD readings from a plate reader as values relative to blank wells containing DMSO only. *P*-value <0.001.(*P*-values were calculated by a two-tailed Student's *t*-test for significance). (**B**) Proliferative effect of CM from OxR HCT116 cells. MTT assay on HCT116 cells treated with CM from parental HCT116 cells (HCT116 CM) or from OxR HCT116 cells (HCT-OxR CM). Cells were either untreated or treated with oxaliplatin as described in the Materials and Methods section. ^*^P-value <0.001.

To test the hypothesis that soluble factors from OxR cells could promote survival and chemoresistance, CM were generated from parental and OxR cells and used to treat parental, chemonaive cells. Figure 1A demonstrates that CM generated by OxR cells was able to stimulate the growth of parental, chemonaive HT29 cells. This effect was also demonstrated in the presence of oxaliplatin (2 *μ*M) and 5-FU (2 *μ*g ml^−1^). Similar results were also observed with CM generated by OxR HCT116 cells when used to treat parental HCT116 cells (Figure 1B). Conditioned media from OxR KM12L4 and OxR RKO cells were also able to stimulate the growth of parental HT29 cells (Supplementary Figure 1).

5-FU-R cells neither generate CM that could stimulate growth of parental cells nor enhance survival in the face of treatment with either oxaliplatin or 5-FU (data not shown). We therefore did not pursue further *in vivo* experiments on these cells as are described below for OxR cells. We did however use CM from 5-FU cells to serve as a second control for our MS analysis of the CM from OxR cells. This provided a control for chronic exposure to cytotoxic agents.

### Effect on bystander cells in mixed cultures

**Figure 2:**
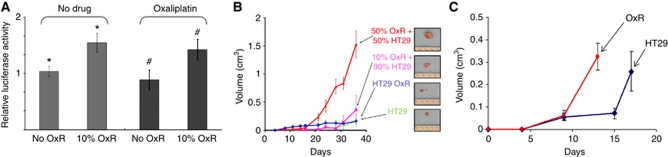
Effect of OxR cells in mixed cultures and mixed tumours. (**A**) *In vitro* mixed-cell experiment. Luciferase activity of ‘bystander’ parental HT29 cells when cultured alone (no OxR cells) or when mixed with 10% OxR cells, either untreated or treated with oxaliplatin. Data are normalised to the control set (no OxR cells). *P*-values were calculated by a two-tailed Student's *t*-test for significance. The symbols ^*^ and # each indicate *P*=0.03 (**B**) In vivo mixed-tumour experiment. Growth curves of *in vivo* tumours composed of four ratios of cells as indicated, with representative photographs of tumours from each cohort. The *y*-axis represents volumes as calculated from caliper measurements. (**C**) Growth curves of secondary tumours in mice with parental HT29 (blue line) and OxR (red line) feeder tumours implanted in the contralateral flank. Days indicate time elapsed after subcutaneous injection of secondary tumours. Feeder tumour sizes of the HT29- and OxR-derived tumours were 0.15 and 0.04 cm^3^ on day 0, respectively, and reached 2.43 and 0.17 cm^3^, respectively, at the termination of the experiment.

To demonstrate that the above-described effect was not an artifact of the generation of CM, cells were mixed to determine the effect of chemoresistant cells on target cells. To specifically assay the effect of the presence of OxR cells on target cells, parental target cells were infected with firefly luciferase, thereby allowing luciferase activity to serve as an assay reflecting the viability/number of target cells only. Figure 2A demonstrates that the presence of OxR HT29 cells at 10% of the total number of cells increased the luciferase activity of target HT29 cells by approximately 50% (*P*=0.03). This effect was also demonstrated in the presence of oxaliplatin (*P*=0.03).

To extend these findings in an *in vivo* system, mixed populations of HT29 cells were injected into the flanks of nude mice in various proportions. Figure 2B demonstrates that mixed tumours grew considerably larger, with the greatest difference observed in tumours consisting of 50% OxR and 50% parental cells. These tumours grew more than six-fold larger than tumours derived from 100% parental or 100% OxR cells (*P*=0.003 and *P*=0.005, respectively). There was no statistically significant difference in the rate of growth of tumours composed solely of parental or OxR HT29 cells in this experiment. Tumours consisting of 10% OxR cells/90% chemonaive cells grew larger than pure counterparts as well, but this difference was not statistically significant. The experiment was terminated due to the size of the 50%/50% mixed tumours, which had reached the IACUC size limits.

### Systemic effect of resistant cells on tumour growth

To demonstrate the systemic effect of soluble factors on tumour growth, we conducted a dual-flank study in which we asked whether a ‘feeder’ tumour that may produce systemic factors could influence the growth of a ‘secondary’ tumour on the contralateral flank. As shown in Figure 2C, secondary tumours in mice with OxR feeder tumours underwent an earlier, accelerated growth phase when compared with secondary tumours in mice with parental feeder tumours. As observed previously, OxR feeder tumours were smaller than parental feeder tumours, a fact that required injection of cells for ‘secondary’ tumours later in mice with OxR feeder tumours. Feeder tumour size of the HT29 and OxR groups were 0.15 cm^3^ and 0.04 cm^3^ on day 0 (day of contralateral tumour cell injection), respectively, and reached 2.43 and 0.17 cm^3^ at the termination of the experiment.

### Conditioned medium from cells enriched for CSCs and its effects on bystander cells

**Figure 3:**
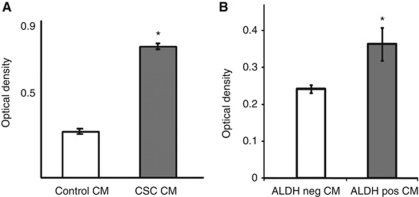
Effect of CM from cultures enriched for CSCs on crc cell proliferation and survival. (**A**) Effect of CM from side-population-based CSCs on growth of HT29 parental cells. Conditioned media were obtained from parental HT29 (control CM) or cells enriched for the CSC phenotype by the side-population assay. These media were used to treat HT29 cells for 48 h, and then MTT assays were performed on triplicate cultures. (**B**) Effect of conditioned media from Aldefluor-based enrichment for CSCs on growth of HT29 parental cells. Conditioned media were obtained from HT29 sorted by the Aldefluor assay for Aldefluor-negative (ALDH neg CM) and Aldefluor-positive (ALDH pos CM) cells. These media were used to treat HT29 cells for 48 h, and then MTT assays were performed on triplicate cultures. ^*^P<0.05.

To determine whether CM from a ‘CSC-enriched’ population of CRC cells enhances growth and survival of CRC cells, we isolated CSCs from parental HT29 cells using two separate functional assays: (1) the ‘side population assay’ based on Hoechst dye exclusion, and (2) the Aldefluor assay based on increased expression and activity of ALDH1A1 (Huang *et al*, 2009). Media conditioned by cells that exclude the Hoechst dye 33342 (the side population) were able to stimulate the growth and survival of parental HT29 cells, as shown in Figure 3A. Figure 3B shows the effect of CM from Aldefluor-positive HT29 cells compared with Aldefluor-negative cells. Media conditioned by Aldefluor-positive cells were able to stimulate growth of parental cells compared with controls (CM from Aldefluor-negative cells). To determine the relative enrichment of chemoresistant cells for the CSC phenotype, the Aldefluor assay was performed on parental, OxR, and 5-FU-R cells. Supplementary Figure 2 shows that OxR cells are significantly enriched for a CSC sub-population relative to parental and 5-FU-R cells.

### Mass spectrometric analysis of CM

To identify potential soluble factors in CM that may have a role in the growth and survival effects of OxR cells, we performed a MS analysis of CM from parental and OxR HT29 cells. As an additional comparison, we also analysed CM from cells made resistant to 5-FU. Conditioned media from 5-FU-R cells had no consistently significant effect on the growth and/or survival of parental cells (data not shown) and thus could serve as a negative control. The analyses yielded tandem MS, from which peptides and proteins were identified. The numbers of spectra that mapped to identified proteins (spectral counts, (Slebos *et al*, 2008)) enabled the generation of a list of proteins present in the CM of OxR cells that were not present (or present in lower amounts) in the CM of both parental and 5-FU-R HT29 cells. Proteins identified were then also analysed using the Ingenuity Pathway Analysis, which allowed us to pare the list down to proteins known to be extracellular/secreted proteins. Table 1 provides a list of those proteins in the OxR CM that satisfied the conditions described above and whose higher expression in the OxR CM was statistically significant, as determined by both a *P*-value as well as a false discovery rate (generated by a modified Poisson analysis as described.) The most significantly upregulated proteins included matrix metalloprotease-7 (MMP7), lipocalin 2, and progranulin.

In order to confirm the expression in human tumour tissues, we analysed data available to the public in the Stanford Microarray Database (Hubble *et al*, 2009) at http://smd.stanford.edu on expression of genes in 9 normal colonic epithelium samples and 15 tumour tissue samples from resected specimens (Saaf *et al*, 2007). Supplementary Figure 3A presents comparative data in terms of log2 fold expression *vs* controls for both normal and tumour samples; the same data is expressed in Supplementary Figure 3B in terms of integer fold expression *vs* controls. Statistically significant (*P*<0.05) differences were found between normal and tumour samples for progranulin, gelsolin, kallikrein 10, lysozyme, and MMP7. For other molecules indicated, expression was not significantly different between normal and tumour samples.

### Phosphoproteomic analysis of CM effect on bystander cells

**Figure 4:**
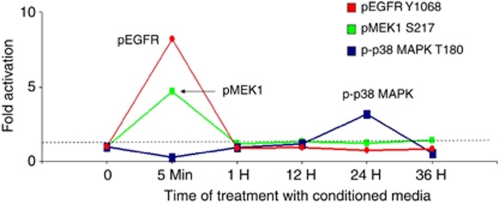
Effect of CM from OxR cells on signalling pathways in target cells: pathway activation mapping analysis. HT29 cells were treated with CM from parental HT29 cells and OxR HT29 cells for times indicated and then analysed by reverse-phase protein microarray. Values are expressed as fold change of protein activation/phosphorylation or expression, as indicated, of cells treated with CM from OxR cells *vs* parental cells. Phosphorylation of EGFR, MEK1, and p38 MAPK are depicted by red, green, and blue graphs, respectively.

To demonstrate the effects of CM from OxR cells on parental HT29 cells, we performed RPMA analysis to map changes in the activation/phosphorylation state in key proteins in target cells involved in known growth and survival signal transduction pathways. Figure 4 demonstrates that the EGFR was found to be rapidly (in 5 min) phosphorylated on tyrosine-1068 in a transient manner. Likewise, MEK1 became phosphorylated rapidly on serine-217, with a similar rapid decline in levels of phosphorylated protein. At a later time point, an increase in total levels of Akt was observed, but no increase in phosphorylated Akt was observed with treatment of cells with CM from OxR cells (Supplementary Figure 4A). Phosphorylation of other molecules was moderately induced by treatment of parental cells with CM from OxR cells *vs* control CM, such as B-Raf, 4E-BP1, mTOR, and GSK (Supplementary Figure 4B), although generally occurring later in the response and with less of a difference in fold activation.

### Effect of progranulin on cell survival

**Figure 5:**
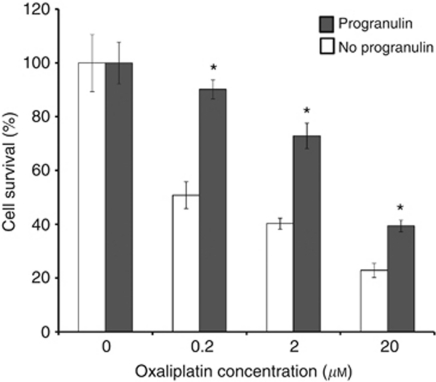
Effect of progranulin on sensitivity to chemotherapy of HT29 colorectal cancer cells treated with oxaliplatin. HT29 cells were treated with or without 1 *μ*g ml^−1^ of recombinant progranulin and oxaliplatin at the indicated concentrations. Cell survival/proliferation was then assayed by MTT assay. Data bars represent the ratio of optical densities of oxaliplatin-treated cells to untreated cells (0) at 96 h for cells treated with progranulin (shaded bars) or cells not treated with progranulin (empty bars). ^*^P<0.05.

In order to demonstrate the effects of progranulin, one of the secreted proteins identified in CM generated by chemoresistant OxR cells, we assayed cell proliferation and survival with the addition of exogenous, recombinant human progranulin. Figure 5 shows that the presence of progranulin resulted in increased survival in the face of oxaliplatin treatment. Treatment of cells with recombinant progranulin enhanced cell survival such that almost twice as many cells were viable after treatment with oxaliplatin. This effect was observed at subtherapeutic oxaliplatin concentration (0.2 *μ*M), peak concentration (2 *μ*M), and at concentrations not achieved clinically (20 *μ*M).

## Discussion

We hypothesised that chemoresistant cancer cells and CSCs generate soluble factors that could mediate growth of otherwise sensitive tumour cells and mediate resistance to chemotherapy. We demonstrated that OxR CRC cells, selected by chronic exposure to high levels of drug, generate soluble factors that can stimulate growth in parental chemonaive CRC cells. This growth-promoting effect made parental chemonaive cells less sensitive to oxaliplatin-induced cell death. Using cell-mixing experiments *in vitro* and *in vivo,* a minority of OxR cells could stimulate the growth of parental cells. In the dual-flank experiment, the presence of a small chemoresistant tumour could have an effect on the growth of a tumour at a distant site, suggesting that resistant cells may exert both a regional and a systemic effect that promotes cancer cell survival. Finally, we showed that cancer cell fractions that are enriched for CSCs also produce soluble factors that mediate growth and resistance, suggesting that sources of soluble factors that promote resistance may be continuously present in the form of CSCs. Several soluble factors were identified that may have clinical significance as indicated by increased expression in tumour specimens over normal tissue. Although the individual roles of these factors are still under investigation, we were able to demonstrate that progranulin may contribute to colorectal cancer cell survival and chemoresistance.

Our results from the *in vivo* study of mixed tumours yielded surprisingly robust results. At baseline, OxR HT29 grew slower than parental HT29 *in vitro* and *in vivo*, hence we did not expect that OxR cells in mixed tumours would overtake parental cells. Despite the fact that OxR cells are themselves slow growing, their presence greatly accelerated growth of mixed tumours, though we were not able to ascertain to what degree the OxR cells or parental cells ultimately contributed to tumour mass. We attempted to label ‘target’ parental cells with dsRed, a modified green-fluorescent protein, in order to assess target cell *vs* OxR cell growth. Unfortunately, expression of dsRed was lost with repeated cell division, hence we could not determine tumour cell content at the end of the *in vivo* study. Although one could consider using luciferase-labelled parental cells to monitor parental cell growth *in vivo*, in a mixed tumour, *in vivo* imaging lacks the resolution to discriminate between the two cell types. These technical considerations aside, taken together with our *in vitro* data, the mixed tumour model supports the concept that OxR cells are able to contribute to a growth-promoting microenvironment.

Important data also comes from the dual-flank study in which tumours were affected in a systemic manner by the presence of another distant tumour composed of resistant cells. Notably, a small distant tumour burden could accelerate the growth of secondary tumours on the contralateral flank of mice-bearing chemoresistant feeder tumours. In this experiment, we introduced a delay in the implantation of secondary tumours in mice in an attempt to control for the different rates of growth of tumours derived from parental cells *vs* OxR cells. The OxR feeder tumours grew at a slower rate than tumours derived from parental cells; in an attempt to control for the slower growth rate of the feeder OxR tumours, we implanted the secondary (parental) tumours at a point when the feeder OxR tumours were smaller that the feeder tumours in the control mice, which were derived from parental cells. Despite this ‘disadvantage’, secondary tumours in the experimental arm (OxR feeder tumours) grew more rapidly in the control arm, despite having a shorter time to grow, and eventually grew larger than the secondary tumours in the control group. From our data, it is not possible to conclude that the effect of the chemoresistant cells is mediated by a simple ‘one-way’ mechanism, but our result shows that even a limited amount of tumour burden with resistant cells may produce a systemic effect of promoting tumour growth and survival at distant sites.

A complex set of molecules are generated by OxR cells, and it is likely that multiple factors function simultaneously to mediate survival and chemoresistance of target cells. Some of the factors identified here are known tumour growth factors. Recently, for example, lipocalin-2 was demonstrated to promote the growth of breast and CRC cells (Hu *et al*, 2009; Yang *et al*, 2009). Our preliminary work suggests that progranulin is a soluble factor that may help mediate the survival of CRC cells under the stress of chemotherapy, and progranulin has been previously reported as a growth and survival factor for several other cancer cell types, such as ovarian (Devoogdt *et al*, 2003; Simpkins *et al*, 2008; Cuevas-Antonio *et al*, 2009), breast (Serrero, 2003; Tangkeangsirisin and Serrero, 2004), and prostate cancer (Pan *et al*, 2004). Oxaliplatin-resistant CM from HT29 contains roughly 30-fold more progranulin than control CM (Table 1), and western blot analysis confirms overexpression of progranulin in OxR CM from HCT116 and KM12L4 cells, although at significantly lower levels (data not shown). The activation of several signal transduction pathways by OxR CM is consistent with previously demonstrated downstream effects of progranulin. MAP kinase, PI3-kinase/Akt, and epidermal growth factor receptor family members have been found to be activated by progranulin (Ong and Bateman, 2003; Monami *et al*, 2006; Cuevas-Antonio *et al*, 2009). Progranulin may mediate its effects through sortilin and/or MET (Hu *et al*, 2010; Li *et al*, 2010). Our data suggests that progranulin may have the ability to mitigate the effects of chemotherapy, and we have data suggesting that recombinant progranulin can function as a growth/survival factor in a dose-dependent manner (data not shown). In other studies, blocking antibodies have been used successfully to inhibit progranulin-mediated activities, but these reagents are not readily available (Kamrava *et al*, 2005; Ho *et al*, 2008).

Identifying the exact source of the activity of OxR CM remains a challenge. Charcoal-based de-lipidation of the CM did not eliminate the activity of OxR CM (data not shown), indicating to us that the activity was not based on the elaboration of such molecules as eicosanoids. We were also not able to identify a specific size fraction that maintained the activity, although generally the activity appeared in all fractions greater than 30 kDa (data not shown). The activity of OxR CM was eradicated by boiling, digesting with trypsin, and high-speed centrifugation to remove a protein pellet (data not shown), suggesting that the activity resides with proteins in the CM. However, we did not address the possibility that nucleic acids, which may be elaborated as free microRNAs or within exosomes, for example, could be mediating the effect of OxR CM. The possibility of exosome-mediated chemoresistance is intriguing in the light of recent data from Higginbotham *et al* (2011) who demonstrated EGFR signalling via exosomes bearing the ligand amphiregulin in colon cancer cells.

Further elucidation of the mechanism of chemoresistance mediated by OxR CM will help identify therapeutic targets. Interestingly, the data presented here suggests that the effect of OxR CM is more pronounced in HT29 cells compared with HCT116 cells (Figure 1). Combined with our data showing activation of the MAP kinase pathway (Figure 4), it is possible to speculate that OxR CM works through the MAP kinase pathway to promote growth and survival. HT29 cells bear the activating V600E mutation in B-RAF, as well as mutations in APC, P53, SMAD4, and PIK3CA. HT29 cells harbour wild-type KRAS, whereas HCT116 cells carry the activating G13D mutation in KRAS, as well as mutations in *β*-catenin, cyclin D kinase 2A, and MLH1. In both cell lines, B-RAF (in HT29) and KRAS (in HCT116) activation appears to drive chemoresistance through activation of MEK 1/2 (Little *et al*, 2011). Our preliminary studies in this regard (not shown) indicate that although the MAP kinase pathway is critical, OxR CM may engage different effector pathways in HT29 and HCT116. This implies that response to therapeutic strategies targeting the MAP kinase pathway might be modulated by the presence of multiple mutations that engages in cross-talk with the MAP kinase pathway.

Our finding that CSCs may also secrete soluble factors that are biologically active suggests that CSCs may also exert systemic effects beyond the local tumour microenvironment. Previous work from our laboratory demonstrated that both OxR and 5-FU-R cells exhibited increased expression of the CSC markers CD133 and CD44 (Dallas *et al*, 2009). Both chemoresistant cell lines also demonstrated functional characteristics of CSCs (i.e., sphere formation and growth in soft agar). Interestingly, OxR cells exhibited a very high percentage of CD133+/CD44+ cells compared with both parental and 5-FU-R cells, suggesting that they were more enriched for the CSC phenotype. 5-FU-R HT29 cells exhibit Aldefluor positivity at frequencies equivalent to parental cells, whereas OxR cells consistently exhibited a higher frequency of Aldefluor-positive cells (data not shown). Given the lower level of expression of CSC markers and the lower level of Aldefluor activity in 5FU-R cells, it appears that 5FU-R cells exhibit less of the CSC phenotype than OxR cells. In the HT29 cell line it was possible to enrich for cells that were more tumourigenic by the Aldefluor assay (data not shown), but this method of separation was imperfect in its ability to exclude tumourigenic cells from the Aldefluor-negative cells. Our data suggests, however, that the Aldefluor assay may be used to enrich for cells with the CSC phenotype, recognising that this is not a pure population of CSCs. The fact that CM from cells enriched for the CSC phenotype by two different methods could stimulate cell growth in parental cells supports a role for CSCs in the tumour microenvironment.

We hypothesise that 5FU-R cells do not generate soluble factors that mediate growth and survival because they are not enriched for the CSC phenotype as compared with OxR cells. Our data supports this hypothesis as OxR cells are enriched for the side population and Aldefluor-positive cells relative to parental controls and 5-FU-R cells, as noted above. Moreover, we consistently observed that OxR and CSC-enriched cells both generate measurable tumours faster than parental cells when implanted in the flanks of nude mice, although these tumours were slower growing than tumours derived from parental cells. It is, however, possible that OxR cells are enriched for a cell subtype that shares CSC characteristics but differs in yet unidentified ways. Similarly, 5-FU-R cells may be enriched in a CSC-like cell type that simply differs from the OxR CSC population. Nevertheless, our data show that cells enriched for the CSC phenotype may function in a similar manner to OxR cells in promoting tumour survival, an activity that may derive from the role of normal stem cells in the maintenance of tissue architecture.

The concept of soluble factors mediating growth and chemoresistance is clinically significant in several settings. From the standpoint of tumour biology, treatment directed at sub-populations of chemoresistant and CSCs may increase disease-free and overall survival by eliminating the source of such factors. In terms of clinical scenarios, such as patients with resectable synchronous metastatic disease, optimal timing of chemotherapy, resection of metastases and resection of primary tumours remains unclear. The presence of soluble, humoral factors that reduce sensitivity of tumours to chemotherapy in some patients may provide a rationale for resection followed by adjuvant chemotherapy in some patients. Similarly, in patients with unresectable metastatic disease, resection of primary tumours that may serve as a source of soluble resistance factors may increase the efficacy of chemotherapy in some patients. The concept that such tumour-derived factors may be induced by exposure to chemotherapeutic agents suggests that these factors could serve as markers of emerging resistance in an individual patient, thereby signalling a need to change chemotherapeutic regimens. Finally, molecules identified, which mediate growth and resistance, as soluble factors may be good targets for therapy with systemic agents.

We have described a novel mechanism of resistance in which cancer cells that become resistant to chemotherapeutic agents may influence the tumour microenvironment; cells that would otherwise be chemosensitive within a tumour, may become chemoresistant due to soluble factors released by chemoresistant/CSCs that influence the survival of these cells through the activation of growth and survival signalling pathways. Furthermore, we found that chemoresistant cells may also exert a systemic effect in animals with multiple sites of disease, suggesting that chemoresistance, like malignant disease itself, may work at the level of the whole organism.

## Figures and Tables

**Table 1:** Proteomic analysis of OxR conditioned media

**Name**	**Log 2 fold**	***P*-value**	**FDR**
Lysozyme (LYZ)	8.080	7.47E-05	0.021
Agrin (AGRN)	7.790	3.25E-04	0.046
Matrilysin (MMP7)	6.508	7.52E-04	0.053
Lipocalin 2 (LCN2)	6.321	5.02E-04	0.048
Serpin peptidase inhibitor clade A (SERPINA1)	5.690	3.15E-05	0.018
Cystatin E/M (CST6)	5.156	1.07E-03	0.061
Granulin (GRN)	5.053	1.52E-03	0.054
Leucine-rich alpha-2-glycoprotein 1			
(LRG1)	4.386	5.91E-03	0.076
Laminin, alpha 5 (LAMA5)	4.205	3.71E-02	0.149
Kallikrein-related peptidase 10 (KLK10)	3.999	5.86E-03	0.078
Semaphorin A (SEMA3B)	2.298	2.52E-02	0.123
Gelsolin (GSN)	2.216	2.74E-03	0.078

Abbreviations: FDR= false discovery rate; OxR=oxaliplatin resistant.

Mass spectrometric analysis of conditioned media from OxR and parental HT29 cells was performed and spectral counts were compared to determine the relative expression levels. Listed here are selected proteins that meet the criteria of being extracellular proteins that are expressed by OxR cells at least 2 log-fold higher than parental cells, have a *P*-value <0.05, and have a false discovery rate <0.20.
