# Supplementary figures and images for: Chemoresistant colorectal cancer cells and cancer stem cells mediate growth and survival of bystander cells
Source: Br J Cancer. 2011 Nov 1;105(11):1759–67. doi: 10.1038/bjc.2011.449 (PMC3242606; doi:10.1038/bjc.2011.449)

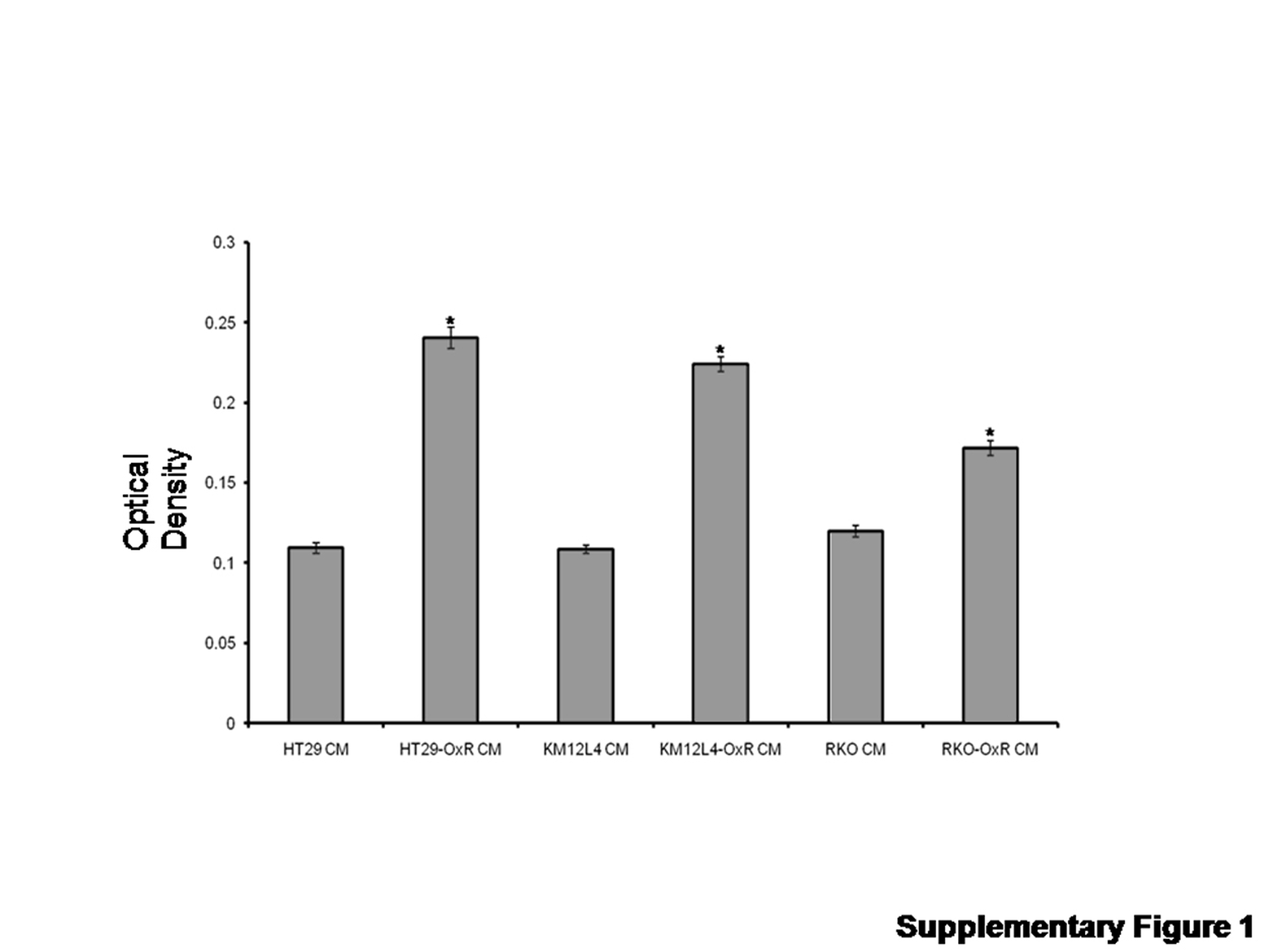

Supplement: Supplementary Figure 1 [file bjc2011449x1.tif]

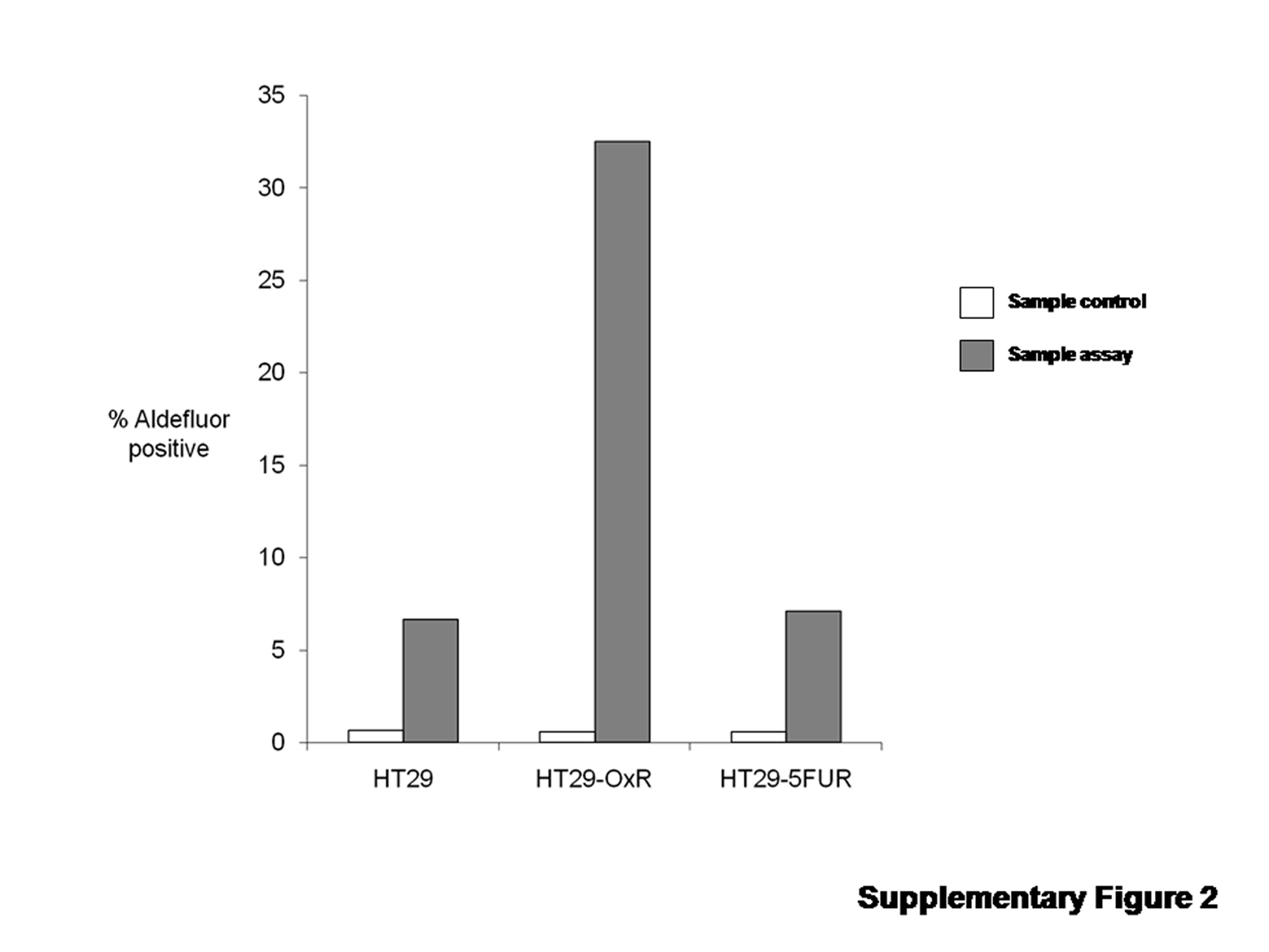

Supplement: Supplementary Figure 2 [file bjc2011449x2.tif]

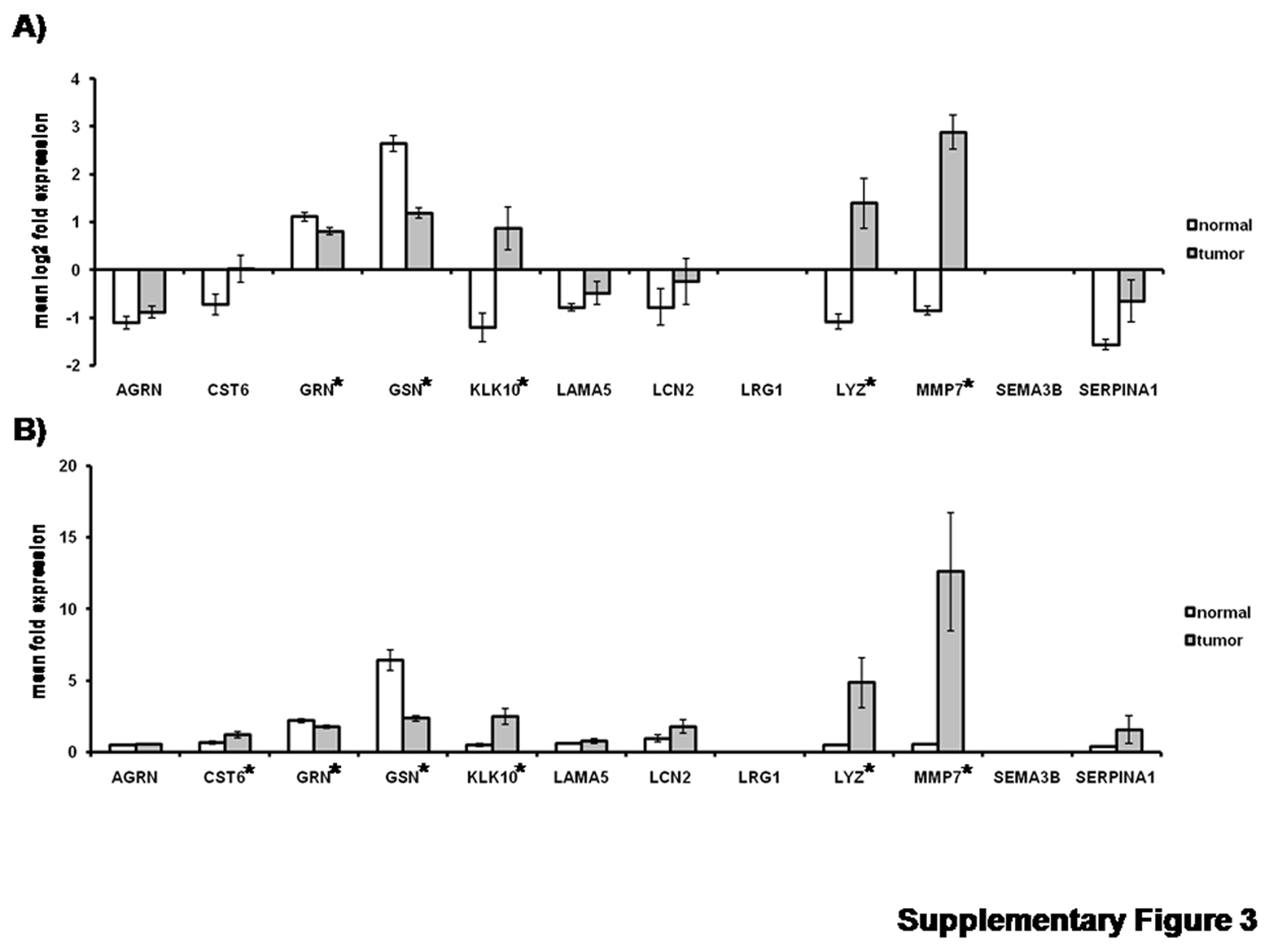

Supplement: Supplementary Figure 3 [file bjc2011449x3.tif]

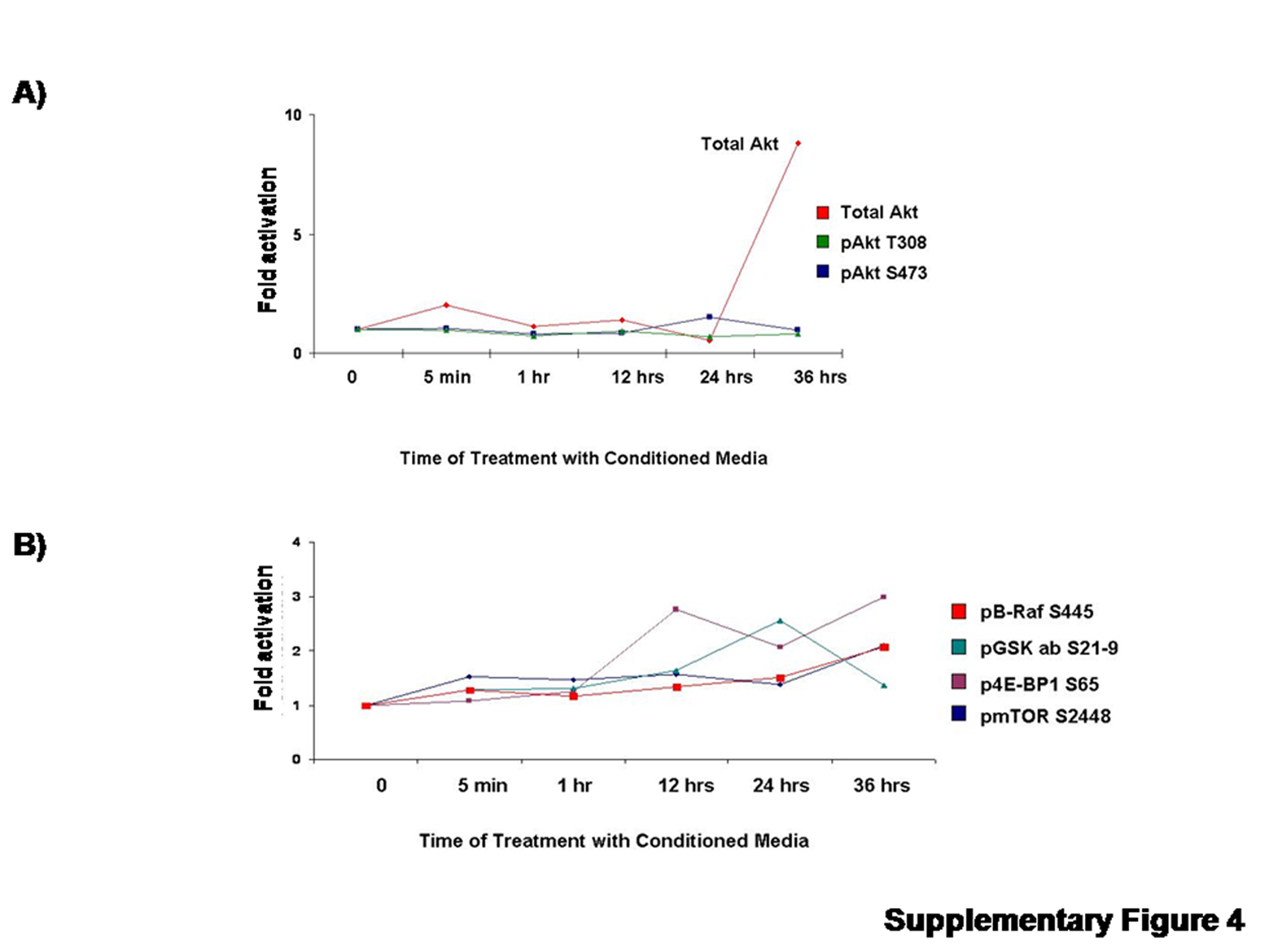

Supplement: Supplementary Figure 4 [file bjc2011449x4.tif]
