# Supplementary material for: Chemoresistant colorectal cancer cells and cancer stem cells mediate growth and survival of bystander cells
Source: Br J Cancer. 2011 Nov 1;105(11):1759–67. doi: 10.1038/bjc.2011.449 (PMC3242606; doi:10.1038/bjc.2011.449)
Supplement: Supplementary Figure Legends [file bjc2011449x5.doc]

**Supplementary Figure 1. Effect of Conditioned Media from Oxaliplatin-Resistant Cells on CRC Cell Proliferation and Survival in Multiple Cell Lines**

MTT assay on HT29 cells treated with conditioned media from parental HT29 cells (“HT29 CM”) or from oxaliplatin-resistant HT29 cells (“HT29-OxR CM”); from KM12L4 or oxaliplatin-resistant KM12L4 cells (“KM12L4 CM” and “KM12L4-OxR CM”); or from RKO and oxaliplatin-resistant RKO cells (“RKO CM” and RKO-OxR CM”). Conditioned media from both KM12L4-OxR and RKO-OxR are able to stimulate the growth of parental HT29 cells. * indicates p<0.05.

**Supplementary Figure 2. Comparison of Cancer Stem Cell Subpopulation in Parental, Oxaliplatin-resistant and 5-Fluorouracil-resistant Colon Cancer Cells**

The Aldefluor assay for ALDH1A1 activity was performed as described on cultures of parental, OxR and 5FU-R HT29 cells cultured under standard conditions. Cells were analyzed by flow cytometry and the results are expressed as a fraction of total cells counted. Clear bars indicate the percent of cells in the sample controls and dark bars indicate the sample result. Results shown are representative of three separate experiments.

**Supplementary Figure 3. Expression of Proteins Identified in Conditioned Media in Microarray Database of Normal Colonic Epithelium and Tumor Tissue**

Expression of the proteins identified in our conditioned media was queried in the Stanford Microarray Database in experiments assessing RNA expression in colonic normal and tumor tissue samples (see text). (A) Mean log2 fold expression values for normal tissue specimens (empty bars) and tumor tissue specimens (shaded bars) were determined for each gene of interest. Genes marked with an asterisk exhibit statistically different expression between tumor and normal tissue. (B) Mean fold expression values corresponding to the data in (A) are presented for normal tissue specimens (empty bars) and tumor tissue specimens (shaded bars).

**Supplementary Figure 4. Effect of Conditioned Media from Oxaliplatin-Resistant Cells on Signaling Pathways in Target Cells: Additional Pathway Activation Mapping Analysis**

HT29 cells were treated with conditioned media from parental HT29 cells and

OxR HT29 cells for times indicated and then analyzed by reverse-phase

protein microarray. Values are expressed as fold change of protein activation/phosphorylation or expression, as indicated, of cells treated with conditioned media from OxR cells versus parental cells. (A) total and phosphorylated Akt, and (B) phosphorylated B-Raf, GSK, 4EBP1, and mTOR.
